# AGAP2: Modulating TGFβ1-Signaling in the Regulation of Liver Fibrosis

**DOI:** 10.3390/ijms21041400

**Published:** 2020-02-19

**Authors:** Amaia Navarro-Corcuera, Eduardo Ansorena, Cristina Montiel-Duarte, María J. Iraburu

**Affiliations:** 1 Department of Biochemistry and Genetics, University of Navarra, 31008 Pamplona, Spain; 2 IdiSNA—Navarra Institute for Health Research, 31008 Pamplona, Spain; 3 The John van Geest Cancer Research Centre, Nottingham Trent University, Nottingham NG11 8NS, UK

**Keywords:** AGAP2, Arf GAP, TGFβ1, liver fibrosis, receptor recycling

## Abstract

AGAP2 (Arf GAP with GTP-binding protein-like domain, Ankyrin repeat and PH domain 2) isoform 2 is a protein that belongs to the Arf GAP (GTPase activating protein) protein family. These proteins act as GTPase switches for Arfs, which are Ras superfamily members, being therefore involved in signaling regulation. Arf GAP proteins have been shown to participate in several cellular functions including membrane trafficking and actin cytoskeleton remodeling. AGAP2 is a multi-tasking Arf GAP that also presents GTPase activity and is involved in several signaling pathways related with apoptosis, cell survival, migration, and receptor trafficking. The increase of AGAP2 levels is associated with pathologies as cancer and fibrosis. Transforming growth factor beta-1 (TGF-β1) is the most potent pro-fibrotic cytokine identified to date, currently accepted as the principal mediator of the fibrotic response in liver, lung, and kidney. Recent literature has described that the expression of AGAP2 modulates some of the pro-fibrotic effects described for TGF-β1 in the liver. The present review is focused on the interrelated molecular effects between AGAP2 and TGFβ1 expression, presenting AGAP2 as a new player in the signaling of this pro-fibrotic cytokine, thereby contributing to the progression of hepatic fibrosis.

## 1. AGAP2: A Unique Member of the Arf GAP Family

Actin cytoskeleton remodeling and membrane trafficking are critical processes for the normal function and survival of the cell. The actin cytoskeleton provides mechanical and structural support to the cell and contributes not only to its internal organization but also to cell division and migration. Furthermore, membrane trafficking is intricately linked to the cytoskeleton, and allows the cell to sort cargo proteins to the correct subcellular compartment through vesicular transport.

Arf (ADP-ribosylation factor) proteins, a subfamily of Ras small GTPases, are key regulators in the control of membrane trafficking and organelle structure and contribute to the remodeling of actin cytoskeleton. In addition, Arf proteins regulate the recruitment and activation of phosphatidylinositol kinases to modulate membrane lipid composition [1]. Their capacity to participate in cell signaling events is determined by the ratio of GTP-bound (active state) to GDP-bound (inactive state) forms. Thus, Arfs are activated by guanine nucleotide exchange factors (GEFs) which catalyze the exchange of GDP for GTP and are inactivated by GTPase-activating proteins (GAPs) which catalyze the hydrolysis of GTP to GDP [2,3,4]. Emerging evidence indicates that an aberrant expression of Arf proteins is associated with several cancers [5,6,7,8,9,10,11,12] therefore, both Arf GEFs and Arf GAPs are considered as novel potential therapeutic targets.

**Figure 1:**
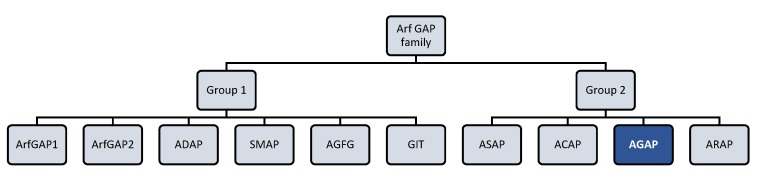
Arf (ADP-ribosylation factor) GTPase-activating proteins (GAPs) family. Human Arf GAP proteins are encoded by at least 31 genes and many of them have multiple splice variants. Structural and phylogenetic analyses have allowed to classify human Arf GAP domain-containing proteins into ten subfamilies termed with a consensus nomenclature. The present figure classifies Arf GAP based on the structural position of the Arf GAP domain. The first group presents the zinc-finger motif (GAP domain) located in the N-terminus of the protein and is formed by ArfGAP1, ArfGAP2, ADAP (Arf GAP with dual pleckstrin homology (PH) domain), SMAP (Amall Arf GAP), AGFG (Arf GAP with XXFG repeats), and GIT (G protein receptor kinase interacting Arf GAP) subfamily members. The second group comprises 21 out of 31 Arf GAP encoding genes and corresponds to Arf GAP proteins containing a tandem of PH domains, Arf GAP and Ankyrin (ANK) repeat domains. It is formed by ASAP (Arf GAP with Src homology 3 domain, ANK repeat and PH domain), ACAP (Arf GAP with coiled-coil, ANK repeat and PH domains), AGAP (Arf GAP with GTPase domain, ANK repeats and PH domain), and ARAP (Arf GAP with Rho GAP domain, ANK repeats and PH domain) subfamily members [13,14].

AGAP2 (Arf GAP with GTP-binding protein-like domain, Ankyrin repeat and PH domain 2) isoform 2 is a protein that belongs to the Arf GAP (ADP-ribosylation factor GTPase activating proteins) family (Figure 1). AGAP2 acts as a GTPase switch for Arf proteins, thereby regulating Arf-mediated signaling. Furthermore, AGAP2 domain-structure enables it to interact also with different proteins and lipids, being involved as a result in different physiological cell functions in an Arf-independent manner.

AGAP2 was first identified [15] and characterized by Chunzhi Xia and colleagues in 2003 [16]. It was initially referred to as GGAP2 (GTP-binding and GTPase-activating protein 2) and described as a bifunctional GTP-binding and GTPase-activating protein, since it was able to bind GTP and unlike other Arf GAP proteins, presented GTPase activity. The structure of the bifunctional multidomain of AGAP2 consists of an N-terminal Ras homology domain, called G domain, followed by the Pleckstrin (PH) domain, the C-terminal Arf GAP domain and the Ankyrin (ANK) repeat domain. It was further demonstrated that, also unlike other Arf GAPs, AGAP2 can activate its GTPase activity via either intermolecular (protein–protein) or intramolecular interactions in a process in which the Arf GAP domain stimulates the G domain, thus enhancing its GTPase activity. Moreover, Chunzhi Xia and colleagues showed that in the absence of the Arf GAP domain, the G domain exhibits a very low GTPase activity, suggesting that the Arf GAP domain is necessary to activate it. This mode of activation is an alternative mechanism for AGAP2 that turns it into a potential and versatile regulator of cell signaling. As other Arf GAP family members, AGAP2 can be also regulated through other mechanisms such as membrane interaction/association via PH domain or protein–protein interaction via ANK repeats [16]. AGAP2 also interacts with Arf proteins and exhibits high affinity for Arf1, Arf5 and Arf6 substrates, in decreasing order of affinity [17].

## 2. Two Human AGAP2 Isoforms: PIKE-L and AGAP2

AGAP2 was described in two independent studies as a centaurin gamma 1 protein, encoded by CENTG1 gene [13,15,18]. Investigations showed that there are two human gene isoforms for AGAP2: AGAP2 isoform 1 which encodes for PI 3-Kinase Enhancer longer isoform L (PIKE-L), and AGAP2 isoform 2 coding for AGAP2, previously known as PIKE-A (Phosphoinositide kinase enhancer-activating AKT) or Cnt-g1 (Centaurin-gamma 1) [13]. These two gene isoforms show high sequence homology, sharing an identical DNA sequence except for their N-terminus. Only the first exon and the first intron are different between these isoforms: The first exon for AGAP2 is located upstream of the first exon for PIKE-L. Thus, the promoter region corresponding to the first exon region of PIKE-L but not AGAP2, seems to be regulated by the selective methylation of a CpG island (ENCODE project). This alternative promoter regulation would explain, at least in part, the fact that AGAP2 isoforms are differentially expressed in tissues: PIKE-L expression is brain-specific while AGAP2 is distributed in different tissues with enriched expression in brain, spleen, heart, small intestine, thymus and peripheral blood leukocytes [16,18,19].

**Figure 2:**
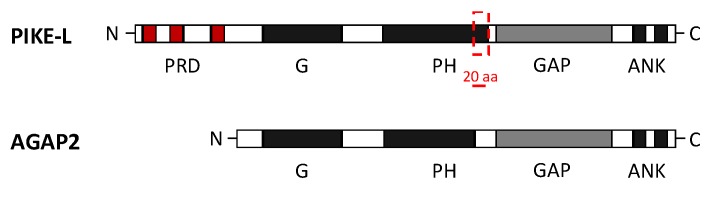
Domain composition of AGAP2 (Arf GAP with GTP-binding protein-like domain, Ankyrin repeat and PH domain 2) and PI 3-Kinase Enhancer longer isoform L (PIKE-L). Both isoforms share an identical structure at the C-terminal but differ at the N-terminal, where they present a GTPase domain (G domain) but AGAP2 lacks the proline-rich domain (PRD). AGAP2 also contains a pleckstrin homology (PH) domain 20 residues shorter than in PIKE-L isoform. The C-terminal structure of both isoforms includes the GTPase-activating proteins (GAP domain) and two ankyrin (ANK) repeats [20].

Both AGAP2 and PIKE-L contain the following domains: N-terminal GTPase domain (G domain), followed by the PH domain, the C-terminal GAP domain and two ANK repeats. PIKE-L, which is a larger isoform, also contains an N-terminal proline-rich domain (PRD). Unlike PIKE-L, AGAP2 isoform contains a shorter PH domain (20 residues shorter than in PIKE-L) and lacks the PRD, due to the alternative promoter usage already mentioned [20] (Figure 2).

The PH domain predominantly binds to different phosphatidylinositol lipids [21] and plays a critical role in mediating subcellular localization and cell signaling [22]. It has been shown that the PH domain of both AGAP2 and PIKE-L exhibits a similar potent binding activity to phosphoinositol lipids. The association of phosphoinositol lipids with the PH domain strongly enhances the GTPase activity of either PIKE-L or AGAP2, exerting stimulatory effects on phosphoinositide 3-kinase (PI3K) and protein kinase B (AKT) signaling, respectively. This interaction also determines the nuclear localization of PIKE-L but not of AGAP2, which primarily resides in the cytoplasm. Sequence analyses revealed that the additional 20 residues contained in the PH domain of PIKE-L are essential for determining the subcellular localization of this isoform (nucleus or cytoplasm) [22]. The PH domain of PIKE-L contains two nuclear signal localization (NSL) motifs: The first one (KRSGNSLNKEWKK) is also present in AGAP2, and the second one (AKRKMWKLKSFGSLRNIYKA) is exclusive for PIKE-L. This second NSL motif keeps PIKE-L in the nucleus upon binding to PI(3,4,5)P_3_ (phosphatidylinositol-3,4,5,-trisphosphate). By contrast, AGAP2 localization is determined only by phosphoinositol lipids which can bind to and affect its GTPase activity [22].

Importantly, it has also been described that PIKE-L binds to PI3K through its N-terminal PRD. Since AGAP2 isoform lacks this PRD domain, it cannot interact with PI3K. Instead, AGAP2 specifically binds to active AKT and up-regulates its kinase activity in a GTP-dependent manner [20].

## 3. Regulation of AGAP2 Expression and Activity

AGAP2 expression has been strongly associated with the progression of tumors and other pathologies [23,24]. Although the aberrant expression of AGAP2 is sometimes due to an amplification of *AGAP2* gene, AGAP2 can be also overexpressed without an alteration in the gene copy number, pointing to other mechanisms taking place.

*AGAP2* promoter and transcriptional activity have been recently characterized. Reporter assays have identified the nucleotides -246/+36 in *AGAP2* DNA sequence containing the minimal *AGAP2* promoter region involved in AGAP2 expression in both chronic myeloid leukemia and prostate cancer human cell lines. Specificity protein 1 (SP1) transcription factor has been found to be bound to this fragment and to be required for AGAP2 expression in these tumoral cell lines [25]. Furthermore, the *AGAP2* promoter fragment -475/-246 contains a functional direct repeat of two motifs (G/AGTTCA) separated by 5 bp (DR5) binding site with a retinoic acid response element (RARE), which seems to be functional as the addition of *all-trans* retinoic acid (ATRA) induced *AGAP2* promoter activity and increased AGAP2 expression. Chromatin immunoprecipitation assays confirmed that the nuclear receptors RARα (retinoic acid receptor α) and RXRα (retinoid X receptor α) and the lysine acetyl transferase acetyl transferase P300/CBP-associated factor (PCAF) were present at the promoter under conditions of *AGAP2* expression in prostate cancer cells. And it was proposed that ATRA might lead to the recruitment of PCAF and perhaps increased SP1 binding to *AGAP2* promoter, thereby leading to increased AGAP2 levels in cancer cells [25].

**Figure 3:**
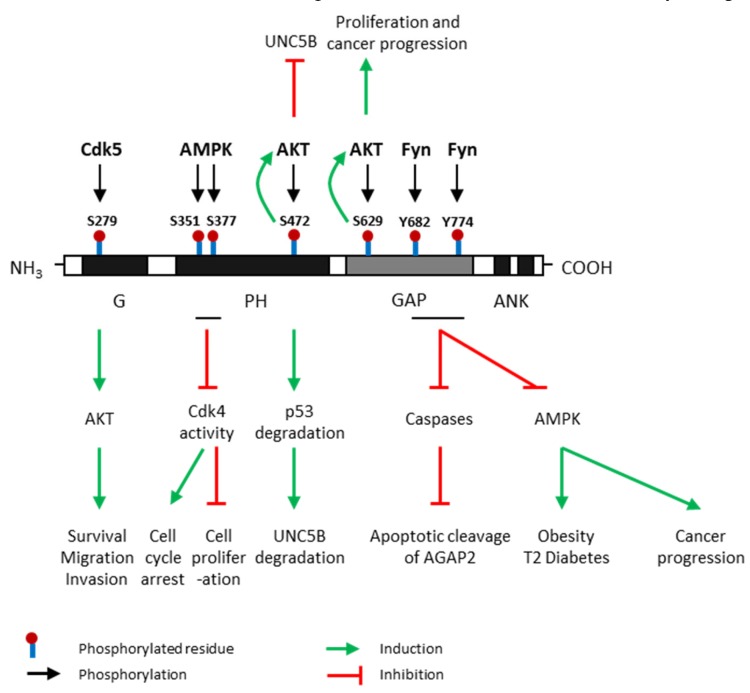
Post-translational modifications of AGAP2 and their effects. AGAP2 (Arf GAP with GTP-binding protein-like domain, Ankyrin repeat and pleckstrin homology (PH) domain 2) expression and activity are regulated by phosphorylation. Cdk5 (cyclin dependent kinase 5) phosphorylates AGAP2 at Serine (S)-279 which is located in its G domain [26]; AMPK (AMP-activated protein kinase), the main energy sensor, phosphorylates AGAP2 on Ser-351 and Ser-377 which are located on its PH domain, under cellular energy stress conditions [25]; AKT (protein kinase B) phosphorylates AGAP2 at S472 [27] and S629 [28] which are in PH and GAP domains, respectively; Fyn phosphorylates AGAP2 at tyrosine (Y) 682 and Y774 in GAP domain [29]. Green arrows indicate process induced by AGAP2 phosphorylated in a precise residue. Red arrows indicate process inhibited by AGAP2 phosphorylated in a precise residue. UNC5B: Uncoordinated-5 netrin receptor B.

Post-translational modifications of AGAP2 regulate AGAP2 expression and activity, and also trigger numerous effects in multiple signaling pathways, modulating cell survival, migration and invasion and contributing to obesity and diabetes development (Figure 3). Several tyrosine and serine residues in AGAP2-containing domains have been proposed as substrate targets for post-translational modifications of AGAP2. These residues are located in three different domains of AGAP2 (G, PH and GAP domains) and can be phosphorylated by, at least, four kinases: Cyclin-dependent kinase (Cdk) 5, AMP-activated protein kinase (AMPK), AKT and Fyn (Figure 3).

G domain – S279: Cdk5 directly phosphorylates AGAP2 at Ser-279 which is located on its G domain, leading to increased GTPase activity. In addition, this phosphorylation further activates AKT kinase activity, promoting cell migration and invasion in human glioblastoma. AGAP2 is considered the first Cdk5 target in cancer cells [26].

PH domain – S351/S377: AMPK, the main energy sensor, phosphorylates AGAP2 on Ser-351 and Ser-377 which are located on its PH domain, under cellular energy stress conditions [30]. Phosphorylation on these residues stimulates the interaction between AGAP2 and 14-3-3β anchor protein promoting the translocation of AGAP2 into the nucleus. Furthermore, AMPK-phosphorylated AGAP2 associates with Cdk4 in the nucleus and inhibits Cdk4 kinase activity inducing cell cycle arrest and the inhibition of cell proliferation. –S472: AKT phosphorylates AGAP2 on Ser-472 which is also located on its PH domain and subsequently enhances its stimulatory effect on AKT activity in a feedback positive loop. AKT-mediated AGAP2 phosphorylation on Ser-472 blocks the uncoordinated-5 homolog B (UNC5B) tumor suppressor inhibiting programmed cell death and triggers p53 degradation in human glioblastoma [27].

GAP domain – S629/Y682/Y774: AKT can also phosphorylate AGAP2 on Ser-629 enhancing AGAP2 GTP-binding activity apparently by downregulating the GTPase activity of the GAP domain. This phosphorylation also stimulates AKT activity increasing cell proliferation and promoting prostate cancer progression [28]. GAP domain contains several tyrosine residues susceptible to being phosphorylated by Fyn which is considered a major physiological tyrosine kinase for AGAP2 [31]. Fyn also binds to and phosphorylates the GAP domain of AGAP2 at two tyrosine residues, Y682 and Y774, protecting AGAP2 from its proteolytic cleavage by active caspases. In addition, it has been demonstrated that Fyn-mediated phosphorylation of AGAP2 promote glioblastoma cell survival as well as adipogenesis and type 2 diabetes development [29]. Interestingly, AGAP2 might be regulated by growth factors since it has been demonstrated that the addition of epithelial growth factor (EGF) triggers AGAP2 phosphorylation on both Y682 and Y774 residues attenuating the apoptotic cleavage of AGAP2 in human cancer [32]. Nevertheless, AGAP2 can be cleaved by the active apoptosome at both D474 (PH domain) and D592 (GAP domain) residues [29].

## 4. TGFβ and AGAP2 in Liver Fibrosis

Liver fibrosis is a pathological condition characterized by an excessive accumulation of extracellular matrix (ECM) proteins in response to persistent liver injury of any etiology, including chronic viral infection, alcoholic liver disease, and non-alcoholic steatohepatitis. Upon chronic damage, the regenerative capacity of the liver is overwhelmed triggering an angiogenic response, sinusoidal remodeling, pericyte (i.e., hepatic stellate cells, HSC) expansion, and altered cell signaling. Progressive fibrosis can eventually result in cirrhosis which is characterized by distortion of the hepatic architecture associated with abnormal blood flow and portal hypertension, subsequent liver failure and/or hepatocellular carcinoma, and is considered the hallmark of chronic liver injury [33,34]. Although several cell types such as portal fibroblasts, bone marrow-cells, hepatocytes, or cholangiocytes are involved in the development of liver fibrosis, HSC constitute the primary cell type responsible for collagen type I synthesis and ECM deposition [35]. Following chronic injury quiescent HSC undergo an activation process and turn into highly proliferative myofibroblast-like cells acquiring contractile, pro-fibrogenic, chemotactic, pro-inflammatory and migratory properties [36]. In addition, this phenotypic change is characterized by the loss of vitamin A droplets, the increase of cytoskeletal proteins like α-Smooth muscle actin (α-SMA), and enhanced ECM protein production. HSC activation is regulated by multiple mediators such as reactive oxygen species (ROS), chemokines, growth factors and cytokines, being the cytokine transforming growth factor beta (TGFβ), the most relevant inducer [33].

TGFβ is considered the most potent profibrogenic cytokine in the liver, and regulates many aspects of ECM structure and function [37]. TGFβ up-regulates the expression of the major ECM proteins such as fibronectin and collagen [38], as well as the expression of cell adhesion protein receptors such as integrins [39], and metalloproteinase (MMP) inhibitors (e.g. TIMPs) [40]. Other components of the ECM, which are less abundant but play relevant roles in the fibrotic liver are also regulated by TGFβ. Tenascin, an ECM protein that is almost absent during normal postnatal life and is expressed in the wound-healing response, is induced by TGFβ, resulting in alterations of cell migration and proliferation [41]. TGFβ also up-regulates basement membrane proteins such as laminin, collagen type IV and entactin [42]. These components are more abundant in the fibrotic liver since the hepatic sinusoids characteristic of normal liver are transformed into continuous capillaries resting on a basement membrane. Besides promoting the fibrotic process, TFGβ inhibits some mechanisms leading to fibrosis reversion. For example, fibrosis can be resolved by apoptosis of HSC caused by natural killer (NK) cells. Interestingly, TGFβ secreted by HSC has been shown by other authors to induce NK cell apoptosis, preventing the clearance of activated HSC (revised in Caja et al. [34]). In the liver TFGβ can be released by different liver cell types including HSC, Kupffer cells, damaged hepatocytes, and Tregs (Regulatory T cells).

The involvement of some Arf GAPs in the pathophysiology of liver cancer has been described [5,43]. However, to date few reports have shown an association with the fibrotic process, even though there is a close relationship between TGFβ1 and Ras small-GTPase signaling. Increasing evidence suggests that the activation of Ras proteins serves as a non-canonical intracellular signal transduction pathway for TGFβ1 [44]. The small-GTPases of the Ras family including Rab, Ran and Rho GTPases are key components in the regulation of TGFβ1 signaling during TGFβ1 receptor endocytosis, Smad trafficking and cross-talk with the actin cytoskeleton, respectively [45,46,47]. A recent review describes the cross-talk between Rho proteins and the TGFβ1 signaling pathway and their implication for the pathogenesis of human diseases as fibrosis [48], and Ren et al. [49] describe how endostatin inhibits TGFβ-induced fibrosis in HSC by modulating the RhoA/ROCK signal pathway.

### 4.1. AGAP2 in TGFβ Receptor Trafficking

TGFβ1 regulates a wide variety of cellular processes in humans, both during development and adulthood [50]. TGFβ1 binds to three different TGFβ receptors (TGFRs), TGFR1, 2 and 3. While TGFBR1 and 2 present serine/threonine and tyrosine kinase activities, TGFBR3 does not have any kinase activity. Upon TGF-β binding, TGFBR2 which is constitutively activated, phosphorylates and activates TGFBR1, resulting in phosphorylation either of SMAD2/3 or noncanonical downstream mediators like extracellular-signal regulated kinase (ERK) or p38 mitogen-activated protein kinase (p38 MAPK). Functional activation of the TGFRs are tightly regulated through integration of post-transcriptional modifications, spatial regulation at the cellular level, and TGFRs availability at the cell surface (recently revised by X. Li et al. [51]). Genetic aberration in TGFRs signaling components have been linked to developmental deficiencies and disorders, whereas alterations in receptor expression and activation are involved in the initiation and progression of fibrosis and cancer [52,53,54]. The bulk of TGFRs resides inside the cells, but the AKT Ser/Thr kinase (AKT) activation in response to insulin or other growth factors rapidly induces transport of TGFRs to the cell surface, increasing the cell responsiveness to TGFβ. Moreover, it has been recently demonstrated that TGFβ itself induces a rapid translocation of its own receptors to the cell surface, amplifying its own response [55]. TGFRs are then internalized and degraded, as a mechanism to end TGFβ1 signaling, or recycled back to the cell surface keeping TGFβ1 signaling activated and thereby maintaining the cellular response to the cytokine [56]. Emerging evidence suggests that TGFR2 trafficking is mediated by different endocytic routes, e.g.: 1) via clathrin when TGFR2 transduce signals and/or is recycled to cell surface; or 2) via caveolae, leading TGFR2 to lysosomal degradation [57]. In the late 1990s, the Smad anchor for receptor activation (SARA) protein was characterized and its function promoting the localization of Smads to TGFRs during TGFβ1 signaling activation was demonstrated. Moreover, this protein was described to be bound to early endosomes involving in membrane trafficking in several cells’ models, including liver cells. The role played by this protein in TGFRs trafficking and TGFβ1 signaling remains controversial, and its expression tends to decrease in liver fibrosis. All these aspects have been recently reviewed by Rozés-Salvador et al. in [58].

**Figure 4:**
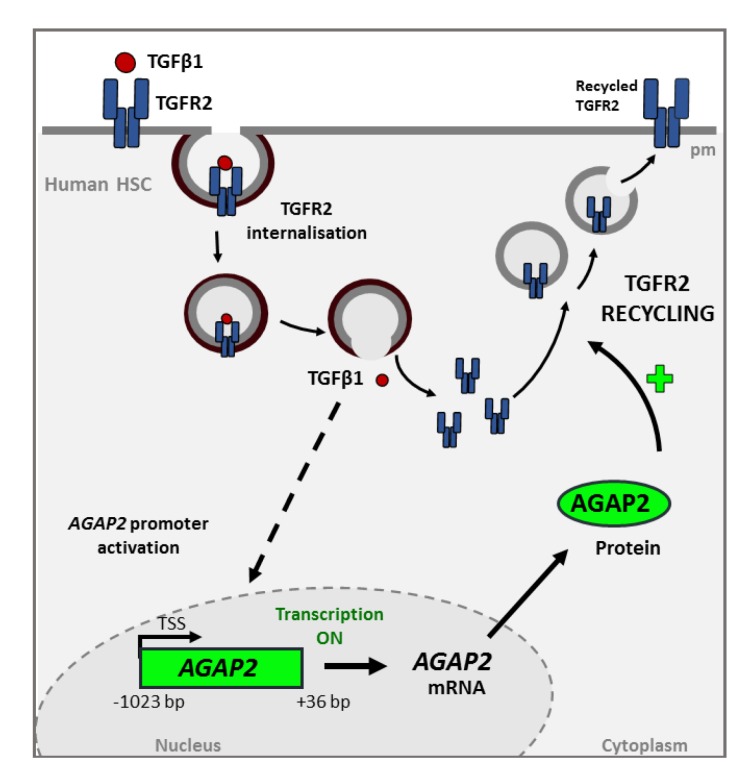
Transforming growth factor beta-1 (TGF-β1)-induced AGAP2 is involved in TGFR2 (TGF Receptor 2) recycling in HSC treated with TGFβ1. The interaction between the cytokine and its TGFR2 receptor triggers the internalization of the complex. This process promotes some signaling cascade whose molecular mechanism have not yet been fully characterized (represented by the dashed line), that induces the transcription and translation of AGAP2 (Arf GAP with GTP-binding protein-like domain, Ankyrin repeat and pleckstrin homology domain 2). AGAP2 is involved in the recycling of the TGFR2 to the membrane and therefore in TGFβ1 signaling. This effect could be either due to a direct interaction between AGAP2 and the receptor or be mediated by the interaction of AGAP2 with other proteins involved in TGFR2 recycling.

Our group described that AGAP2 silencing altered the recycling of TGFR2 back to the plasmatic membrane in the human osteosarcoma cell line U2OS and the HSC LX-2 human cell line [59] (Figure 4). AGAP2 knocked-down cells showed TGFR2 accumulated in the perinuclear region whereas control cells presented TGFR2 distributed throughout the cell. This effect could be either due to a direct interaction between AGAP2 and the receptor or may be mediated by the interaction of AGAP2 with other proteins involved in TGFR2 recycling such as Rab proteins (e.g., Rab 11) [60]. A role of AGAP2 in receptor trafficking is not exclusive of TGFBR2. Unpublished data from our own group, showed that, in U2OS cells, AGAP2 regulates the trafficking of glucose transporter 4 (GLUT4) induced by incubation with insulin. GLUT4-mediated glucose-uptake requires the formation of GLUT4-storage vesicles (GSV), their translocation from the perinuclear region to the cell surface, and GSV fusion with plasma membrane. Silencing AGAP2 in insulin-treated U2O2 cells caused an abnormal condensation and accumulation of GLUT4 and diminished the presence of membrane-fused GLUT4.

The effect of AGAP2 in receptor trafficking has been also described for other receptors. For example, AGAP2 is involved in promoting the fast recycling of transferrin receptor by co-localizing with clathrin adaptor protein (AP1) and Rab4 in endosomes [61], and also regulates the retrograde transport between early endosomes and the trans-Golgi network by interacting with Rab7 [62]. Moreover, AGAP2 forms a complex with β-arrestin 2 to promote the recycling of internalized β2-adrenergic receptor (β2-AR) receptor, a G-protein-coupled receptor involved in inflammation and immunoregulation. These β2-AR receptors are controlled by proteins such as β-arrestins, which regulate β2-AR endosomal internalization through clathrin-coated vesicles [63,64]. AGAP2 regulates β-arrestin 2 membrane association as AGAP2 knock-down disrupted β2-AR trafficking and prevented β2-AR recycling back to the membrane. As a consequence, β2-AR accumulated in endosomes in the perinuclear region in the cell. In addition, AGAP2 also forms a complex with ERK and stimulates the β2-AR-induced ERK phosphorylation. Thus, AGAP2 also plays a regulatory role in β-arrestin-dependent signaling of β2-AR [65].

### 4.2. TGFβ1 and AGAP2 in HSC Proliferation and Migration

**Figure 5:**
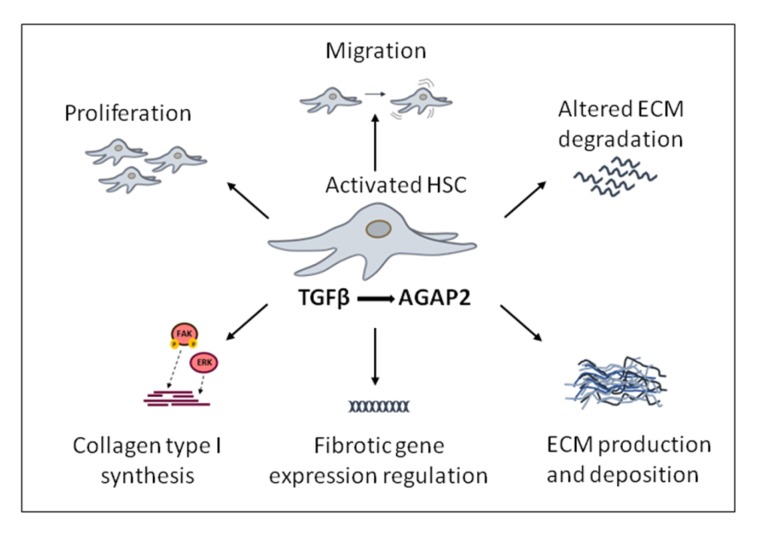
Transforming growth factor beta (TGFβ)-induced pro-fibrotic effects modulated by AGAP2 (Arf GAP with GTP-binding protein-like domain, Ankyrin repeat and pleckstrin homology domain 2) in activated hepatic stellate cells, (HSC) cells.

Enhanced cell migration and proliferation are characteristic of activated HSC and can be potentiated by TGFβ1 through different mechanisms. Our group found that both proliferation and migration in response to TGFβ1 were diminished in activated HSC in which AGAP2 expression had been previously silenced (Figure 5). As previously mentioned, the main effect triggered directly by TGFβ1 in HSC is to induce the transdifferentiation of quiescent HSC into activated myofibroblasts rather than proliferation or migration [66]. By contrast, platelet-derived growth factor 2 (PDGF) is the master mitogenic cytokine promoting proliferation of HSC after liver injury [67]. Interestingly, there is evidence showing that TGFβ1 promotes HSC proliferation by activating PDGFβ expression pointing to a convergence of PDGFβ and TGFβ1 signaling for HSC activation [66,68]. TGFβ1 induces an increase on PDGFβ mRNA levels, in a process that is mediated by AGAP2 expression in HSC. Thus, TGFβ1 might be inducing HSC proliferation by increasing PDGF in an AGAP2-dependent manner [59].

Together with proliferation, migration of activated HSC contributes to fibrosis. Cell migration is a dynamic and multistep process of leading-edge protrusions, turnover of focal adhesion, generation of traction forces, and tail retraction and detachment [69,70,71]. After liver damage, TGFβ1 and other growth factors such as EGF initially lead to a spread of HSC within the space of Disse. Afterwards, chemoattractive stimulation from TGFβ1 promotes further migration of HSC into the liver injured areas where ECM components are accumulated, perpetuating fibrosis progression [67,72]. Our group also found a positive effect of TGFβ1 in migration of HSC that was proliferation independent and mediated, at least in part, by AGAP2. The stimulatory effect of AGAP2 on TGFβ1-induced HSC migration could involve different molecular mechanisms. In this sense, AGAP2 has been recently described as an Arf GAP which regulates integrin adhesion complexes by binding to and regulating focal adhesion kinase (FAK) [45,65]. This protein was named based on its prominent localization in the focal adhesions [73], cellular structures that connect actin cytoskeleton with the ECM formed by: 1) signaling proteins such as FAK, paxillin, and c-Src; and 2) structural proteins as talin and vinculin [74]. They are constantly disassembled and remodeled in a dynamic fashion [75,76]. The interaction between Arf GAP proteins and FAK in the process involved in the focal adhesion assembly had been previously described [77]. Zhu et al. demonstrated that following EGF receptor stimulation, AGAP2 forms a complex through its PH domain with FAK, increasing its kinase activity, inducing the focal adhesion disassembly that lead to increased cell migration [78]. Moreover, Dwane et al. showed that AGAP2 binds to the scaffolding protein Receptor of activated protein kinase C1 (RACK1) and is scaffolded to FAK, regulating FAK activity and promoting neurite outgrowth in brain tissue [79]. On the other hand, recent reports also show that TGFβ can activate kinases involved in actin remodeling (e.g. FAK). Particularly, TGFβ-activated FAK increases α-SMA and collagen production in HSC and promotes cytoskeletal reorganization during myofibroblast differentiation [80].

Interestingly, AGAP2 overexpression up-regulates FAK activity and, AGAP2 knock-down decreases the activity of FAK. It seems that this AGAP2-induced FAK activity involves AGAP2 N-terminal G domain but is independent of the GAP domain. Furthermore, Xue-Ke Zhao et al. have shown that the inhibition of FAK activation significantly reduces HSC migration and small-GTPase activation in PDGF-treated HSC [80]. Therefore, AGAP2 binding partner FAK, could also be responsible for the effects in migration of HSC in response to TGFβ1.

Cell migration and proliferation are features of activated HSC and can be potentiated by TGFβ1 through different mechanisms (Figure 5). Emerging evidence suggests that TGFβ, through increased fibronectin trafficking, is a direct inducer of the fibrillogenesis required for TGFβ-induced cell migration. In response to TGFβ, internalized fibronectin is not degraded by lysosome but instead is recycled for fibrillogenesis, a process dependent of the TGFR2 [34,81].

### 4.3. AGAP2 in TGFβ1-Induced Fibrogenesis on HSC

The role of AGAP2 modulating the pro-fibrogenic effects induced by TFGβ1 in liver fibrosis also includes gene regulation processes. Pro-fibrotic genes whose expression is induced or inhibited by TFGβ in HSC cells, depending at least in part, on AGAP2 expression have been identified [59]. More precisely, among all these TFGβ-induced pro-fibrotic genes, a direct relationship between AGAP2 and collagen type I levels, the major ECM component of the fibrotic tissue was found (Figure 5). Depletion or increased expression of AGAP2 correlated with diminished or enhanced collagen type I levels in response to TGFβ1 in the same cell type. In this sense, AGAP2 was regulating FAK phosphorylation in activated HSC, and both FAK and ERK were mechanisms underlying this AGAP2-mediated collagen type I expression in HSC treated with TGFβ1 [59]. In agreement with these results, Y. Zhu et al. had previously reported that AGAP2 interacts with and regulates FAK activity in both HEK293 and U87 cell lines [78]. In addition, Yuanjun Wu et al. demonstrated that AGAP2 forms a complex with endogenous ERK and that AGAP2 overexpression potentiates ERK phosphorylation induced by β2-AR [65]. Thereby AGAP2 would be also promoting the progression of liver fibrogenesis.

### 4.4. TGFβ1 Induces AGAP2 in HSC

TGFβ1 has been described as a positive regulator of AGAP2 expression in activated HSC. Both the activity of AGAP2 promoter as well as protein levels of AGAP2 were enhanced by treatment with TGFβ1. These results were validated in a pathophysiological context, where AGAP2 levels were also significantly increased in liver samples obtained from thioacetamide (TAA)-treated rats that presented a high degree of fibrosis [59]. Interestingly, *SP1* which binds to *AGAP2* promoter as indicated before, is highly expressed by activated HSC [82] and is a potent transcriptional factor that mediates cell proliferation and regulates fibrotic genes on activated HSC [83]. In addition, TGFβ1 increases SP1 expression on HSC [84]. Thus, *AGAP2* promoter activity could be regulated by *SP1* in activated HSC in a TGFβ1-dependent or -independent manner.

## 5. Conclusions and Prospects

The discovery of the Arf GAP AGAP2 less than 20 years ago triggered a substantial amount of work trying to elucidate the pathophysiological roles of this GTPase/GTP-activating protein and its mechanisms of action. However, questions regarding mechanisms that regulate AGAP2 expression and an exhaustive list of pathways affected by AGAP2 expression remain unanswered. Importantly, the fact that AGAP2 expression has been shown to be modulated by ATRA and TGFβ1, suggests that AGAP2 activation might depend on lipids and growth factors in a specific manner. The possible role of epigenetics, miRNAs, or lncRNAs in the regulation of AGAP2 expression needs also to be explored. *AGAP2-AS1,* a newly identified lncRNA located in the 3’ region of the AGAP2 gene that also promotes cell proliferation, migration, invasion, EMT progression and inhibition of apoptosis in hepatocellular carcinoma cells in vitro and in vivo [85], might be perhaps regulating AGAP2 expression.

Most of the studies on AGAP2 aimed to determine the roles of AGAP2 and describe the effects of increased levels of AGAP2 in different cellular contexts. Thus, we now know that AGAP2 is involved in different functions including cell proliferation and survival, remodeling of actin cytoskeleton, or membrane trafficking, and that these functions turn AGAP2 into a potential target in different pathologies such as cancer and fibrosis. In this sense, the present article has described how AGAP2 mediates at least in part some of the pro-fibrogenic effects described in HSC cells by the master pro-fibrotic cytokine TFGβ. These findings point to a positive regulatory loop between AGAP2 and signaling molecules triggered by TGFβ1. Since TGFβ1 strongly promotes pro-fibrogenic effects in other organs such as lung and kidney [80,86,87], a role for AGAP2 in TGFβ1-mediated pro-fibrogenic effects in other fibrotic organs should be explored. Further studies would help to determine the extent and relevance of these effects and therefore the possibility of AGAP2 being a molecular target for fibrosis.

## Abbreviations

α-SMA	α-Smooth muscle actin
AGAP2	Arf GAP with GTP-binding protein-like domain, Ankyrin repeat and PH domain 2
AKT	AKT serine/threonine kinase, also known as ‘protein kinase B’
AMPK	AMP-activated protein kinase
ANK	Ankyrin
AP	Adaptor protein
Arf	ADP-ribosylation factor
Arf GAP	ADP-ribosylation factor GTPase activating proteins
ATRA	all-trans retinoic acid
β2-AR	β2-adrenergic receptor
Cdk	Cyclin-dependent kinase
Cnt-g1	Centauring-gamma 1
DR5	Direct repeat (DR) of two motif (G/AGTTCA) separated by 5 bp
ECM	Extracellular matrix
EGF	Epithelial growth factor
ERK	Extracellular-signal-regulated kinase
FAK	Focal adhesion kinase
GAPs	GTPase-activating proteins
GEFs	Guanine nucleotide exchange factors
GLUT4	Glucose transporter type 4
GSV	GLUT4-storage vesicles (GSV)
HSC	Hepatic stellate cells
MMP	Metalloproteinase
NK	Natural killer cells
NSL	Nuclear signal localization
PCAF	Acetyl transferase P300/CBP-associated factor
p38 MAP	p38 mitogen-activated protein kinase
PDGF	Platelet-derived growth factor 2
PH	Pleckstrin homology
PI3K	Phosphoinositide 3-kinase
PIKE-L	PI 3-Kinase Enhancer longer isoform L
PRD	Proline-rich domain
Rab	Ras-associated binding protein
RACK1	Receptor of activated protein kinase C1
RARE	Retinoic acid response element
SARA	Smad anchor for receptor activation
SP1	Specificity protein 1
TAA	Thioacetamide
TIMP	Tissue inhibitor of metallopeptidases
TGFR	Transforming growth factor β receptor
Treg	T regulatory cells
UNC5B	Uncoordinated-5 netrin receptor B
